# Electrical Stimulation with a Conductive Polymer Promotes Neurite Outgrowth and Synaptogenesis in Primary Cortical Neurons in 3D

**DOI:** 10.1038/s41598-018-27784-5

**Published:** 2018-06-29

**Authors:** Qingsheng Zhang, Stephen Beirne, Kewei Shu, Dorna Esrafilzadeh, Xu-Feng Huang, Gordon G. Wallace

**Affiliations:** 1 Intelligent Polymer Research Institute, ARC Centre of Excellence for Electromaterials Science, AIIM Facility, Innovation Campus, University of Wollongong, Squires Way, Fairy Meadow, NSW 2519 Australia; 2 Illawarra Health and Medical Research Institute, Wollongong, NSW 2522 Australia; 3 Present Address: Centre for Advanced Electronics and Sensors (CADES), School of Engineering, RMIT University, Melbourne, VIC 3000 Australia; 4 Centre for Translational Neuroscience, School of Medicine, University of Wollongong, Wollongong, NSW 2522 Australia

## Abstract

Deficits in neurite outgrowth and synaptogenesis have been recognized as an underlying developmental aetiology of psychosis. Electrical stimulation promotes neuronal induction including neurite outgrowth and branching. However, the effect of electrical stimulation using 3D electrodes on neurite outgrowth and synaptogenesis has not been explored. This study examined the effect of 3D electrical stimulation on 3D primary cortical neuronal cultures. 3D electrical stimulation improved neurite outgrowth in 3D neuronal cultures from both wild-type and NRG1-knockout (NRG1-KO) mice. The expression of synaptophysin and PSD95 were elevated under 3D electrical stimulation. Interestingly, 3D electrical stimulation also improved neural cell aggregation as well as the expression of PSA-NCAM. Our findings suggest that the 3D electrical stimulation system can rescue neurite outgrowth deficits in a 3D culturing environment, one that more closely resembles the *in vivo* biological system compared to more traditionally used 2D cell culture, including the observation of cell aggregates as well as the upregulated PSA-NCAM protein and transcript expression. This study provides a new concept for a possible diagnostic platform for neurite deficits in neurodevelopmental diseases, as well as a viable platform to test treatment options (such as drug delivery) in combination with electrical stimulation.

## Introduction

Deficits in neurite outgrowth and synaptogenesis have been recognized as an underlying developmental aetiology of psychosis^1^. Unfortunately, traditional pharmacological intervention has limited efficacy in treating neurite outgrowth deficits^2^, and is usually associated with haematological and metabolic side effects^3–5^. Recent studies suggest that electrical stimulation can improve neurite outgrowth^6–9^, and may therefore become a novel treatment option for cognitive deficits in neurodevelopmental and neurodegenerative diseases, including schizophrenia.

We have previously shown that electroactive polypyrrole (PPy) doped with dodecylbenzenesulfonic acid (DBSA) promotes neuronal induction, including neurite outgrowth and branching in primary cortical neuronal cultures from normal and disease model animals^10,11^, as well as in human neural stem cells^12^. However, these applications are based on a two-dimensional (2D) cell culture system, while neural tissues are actually three-dimensional (3D) structures^13^. The importance of examining cell behaviour using 3D as opposed to 2D structure has been highlighted in a number of recent important studies^14–16^. Therefore, a 3D electrical stimulation system is required to test the effect of electrical stimulation on 3D cell cultures.

Electrical stimulation has been reported to induce enhanced neurite outgrowth in 3D cell culture^6^. However, the effect of 3D electrical stimulation from 3D electrodes on neurite outgrowth and synaptogenesis has not been explored. An interdigitated 3D electrode system has previously been developed and reported by our group^17^, but its application on biological systems has not been investigated.

Here, we described the design and fabrication of an interdigitated 3D electrode system and examined the effect of 3D electrical stimulation delivered by these electrodes on 3D primary cortical neuronal cultures from normal and disease model mice. We also compared the differential effect of electrical stimulation on neurite growth, synaptogenesis, and cell migration between 2D and 3D. Finally, we tried to unravel a potential molecular biological mechanism that may underlie these effects.

## Methods

### Fabrication of 3D interdigitated electrodes

Designs for two separate metal electrodes and a polymer spacer were prepared in Solidworks computer aided design software. The volume of the designed cell was minimised for efficient use of cell materials and to ensure compatibility with microscopy systems. The spacer elements to ensure electrical isolation of the electrodes was produced using an Objet Connex 350 (Stratasys, USA) in a biocompatible proprietary material, MED610 (Stratasys, USA). Electrodes with nine pillars, each with diameter 300 µm and height of 3 mm were produced in Ti6Al4V alloy (TLS TechnikSpezialpulver, Germany) using a Realizer SLM50 (Realizer GmbH, Germany). Printed polymer components were removed from the build tray and support material manually removed prior to cleaning as per Stratasys protocol. Printed metal electrodes were manually removed from the build tray using side cutters and supported surfaces manually finished with 600 grit sandpaper. Electrodes were then sonicated in IPA for 30 mins to remove any loosely bound metal particles.

### Coating Process

The Pyrrole (Py) monomer and the dopant, DBSA were obtained from Sigma-Aldrich (Sydney, Australia). The Py was distilled prior to use. All Py monomer and dopant solutions were prepared with deionized water (Milli-Q). Prior to electrochemical deposition, printed 3D interdigitated electrodes were cleaned by sonication in acetone and deionized water. PPy was deposited on 3D electrodes using potentiostatic electropolymerization method. The electrodeposition was conducted using an Ag/AgCl reference electrode and a stainless steel mesh counter electrode, in aqueous solution containing 0.1 M pyrrole and 0.05 M DBSA with 0.75 V applied potential for 20 min. The electrodes were then rinsed with water and dried in a vacuum oven overnight at 50 °C. The choice of conductive polymer as coating material was based on our previous experiments in 2D^10,11^.

**Figure 1:**
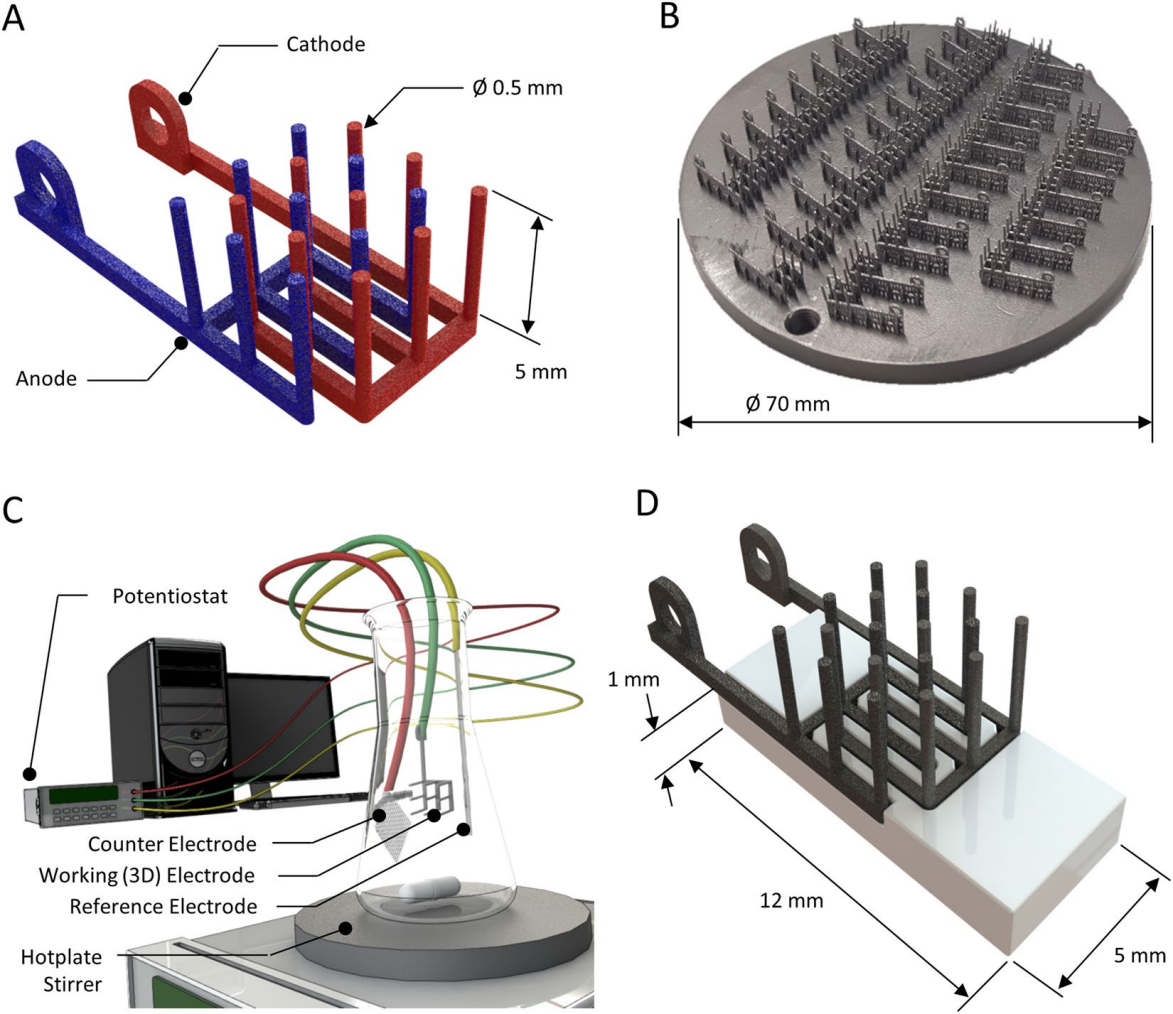
3D interdigitated electrode design and production process. (**A**) Schematic representation of two 3D electrode units in an interdigitated arrangement – pillar dimensions provided; (**B**) Batch quantity of TiAl6V4 electrode pairs as produced by selective laser melting; (**C**) Render representation of electropolymerisation coating process; (**D**) Render of coated electrodes assembled into 3D printed frame to maintain electrode spacing throughout cell stimulation studies – dimensions provided to indicate scale of complete 3D electrode pair assembly.

Interdigitated electrode arrangements (Fig. 1) were manually assembled by bonding coated Ti6Al4V electrodes to the spacer by the addition of MED610 and curing by application of UV light for 30 seconds. The selection of Ti6Al4V for our electrodes is because: (1) it is 3D printable; (2) it is suitable for conducting polymer coating. The assembled structure was then thoroughly cleaned in water, followed by immersion in 2% wt/vol sodium hydroxide in water for 2 hrs, rinsing in water, before immersion in IPA for 30 mins and then allowed to dry in air prior to use.

**Figure 2:**
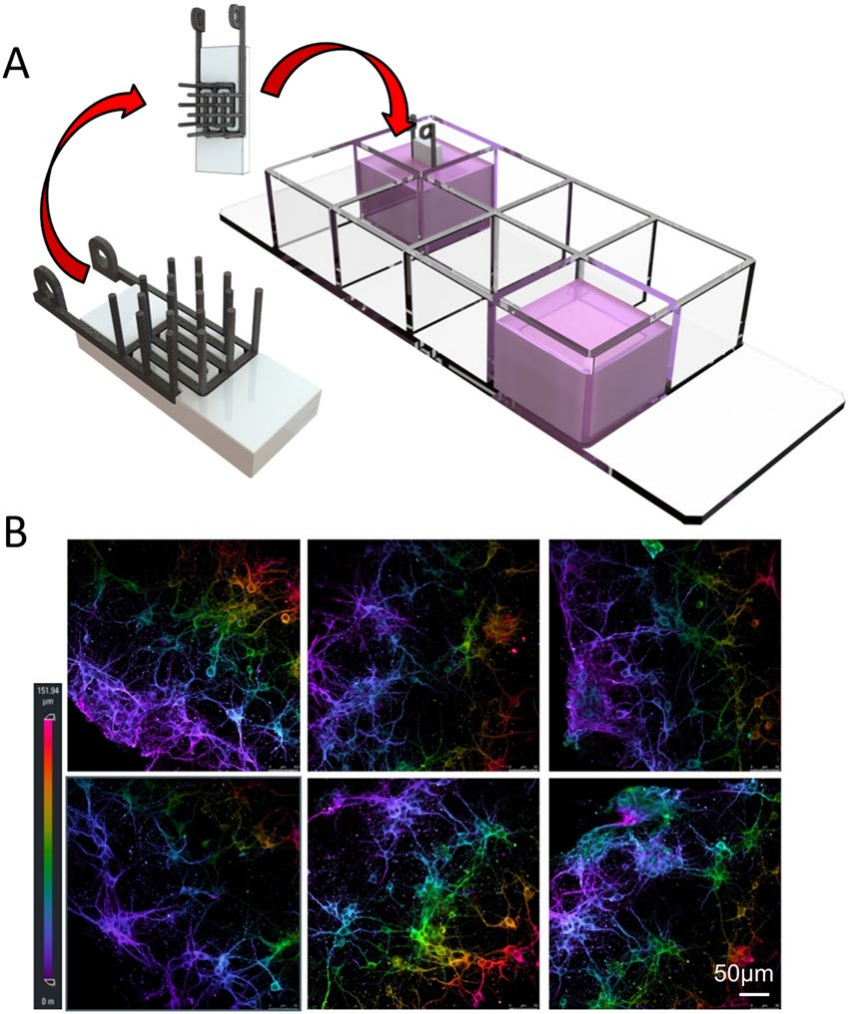
3D interdigitated electrical stimulation setup. (**A**) Schematic illustration of the 3D interdigitated electrodes and cell culture setup; (**B**) 3D immunofluorescence images of the 3D primary neuronal cultures in collagen. Scale bar = 50 µm.

The dimensions of the designed electrode constructs are demonstrated in Fig. 1D. A schematic illustration of the process steps is demonstrated in Fig. 1, and a picture showing the stimulation chambers and the electrodes is in Fig. 2A, while Fig. 2B shows a sample immunofluorescence image of 3D primary neuronal cultures encapsulated with collagen in this device.

### 3D primary prefrontal cortex (PFC) neuronal cultures in collagen

Experiments were run with 6 parallel samples and repeated 3 times. The heterozygous NRG1-KO mice^18^ have been described previously. Heterozygous NRG1-KO male and wild-type C57BL/6 J female mice were mated to obtain either heterozygous NRG1-KO or wildtype pups. All experimental procedures were approved by the Animal Ethics Committee, University of Wollongong, Australia, and complied with the Australian Code of Practice for the Care and Use of Animals for Scientific Purposes^19^.

On postnatal day 0 (PN0), genotypes were determined by tail biopsy and polymerase chain reaction (primers for Nrg1-KO mice: Neo173F 5′-ATGAACTGCAGGACGAGGCA-3′ and Neo6301R 5′-GCCACAGTCGATGAATCCAG-3′; primers for wild-type mice: 5′-AACAGCCTGACTGTTAACACC-3′ and 5′-TGCTGTCCATCTGCACGAGACTA-3′. Primary pre-frontal cortical cultures were prepared as described previously^20^. In brief, postnatal day 0 (PN0) pups were decapitated and brains were dissociated aseptically. Brains were then placed into ice cold dissecting solution. After careful removal of the meninges and superficial blood vessels, the prefrontal cortex area from both hemispheres was dissected and cut into approximately 1 mm pieces. The tissue pieces were incubated at 37 °C for 30 mins in papain-containing enzyme solution. Digested tissue pieces were washed in solutions containing enzyme inhibitors, and then dissociated by triturating in neurobasal media (NBM) (Sigma-Aldrich, Sydney, Australia) through glass pipettes and collected in warm NBM supplemented with B27 (Sigma-Aldrich, Sydney, Australia). Dissociated cortical cells were encapsulated in 3D collagen culture kit (Merck Millipore, VIC) at a final density of 1 × 10^6^ cells/ml and plated over the interdigitated electrodes coated with polypyrrole polymers (PPy.DBSA). This cell density was selected based on our preliminary condition tests. For 3D cell culture, the cell density is significantly higher than those in 2D. There might be variations for proximity of cells from sample to sample, but this has been captured by increasing the overall sample size during the data collection process.

5-FDU was added to the conditional NBM (which was used to replace the NBM) to halt the growth of non-neuronal cells. Cultures were maintained at 37 °C in a humidified CO_2_ incubator with media changed every 3 days. Cells were used for experiments for 7 days *in vitro* (DIV7).

### 3D electrical stimulation

Electrical stimulation was applied to corresponding groups of cell culture in accordance with the clinically relevant published method^21^. Briefly, cells were stimulated for 8 h per 24 h period for 3 days under 5% CO_2_ humidified atmosphere at 37 °C. The electrodes were fabricated with 3D printing technique (as detailed above), then coated with or without Ppy.DBSA. The cells were stimulated at 1 ± 0.25 mA/cm^2^ using a biphasic waveform of 100 μs pulses with 20 μs interphase open circuit potential and a 3.78 ms short circuit (250 Hz) using a Digital StimulatorDS8000 and A365 Isolator units (World Precision Instruments) interfaced with an e-corder system (eDAQ). The voltage waveform across the active electrode area in response to the applied current pulse was recorded.

### Immunofluorescence

Cells were grown on Control or Ppy-DBSA coated electrodes until DIV7, when electrical stimulation (8 hours per day, for 3 consecutive days) was conducted (for groups without electrical stimulation, wells were placed next to the stimulation groups so that the cells had the same growth conditions as those in the stimulation groups) before being fixed in 3.7% paraformaldehyde. Cells were then washed in PBS, and permeabilized with 0.3% Triton X-100 in PBS for 10 min. After blocking with 5% normal goat serum for 2 h at room temperature, primary antibody incubations were performed in 1% goat serum in PBS overnight at 4 °C, followed by incubation in a secondary antibody cocktail of Alexa Fluor 488, 594, and DAPI (Thermo Fisher Scientific, NSW) for 2 h at room temperature. Cells were viewed using a 40× or a 63× dry objective on a DMI6500B confocal microscope (Leica, Mannheim, Germany). ImageJ 1.46r with NeuriteQuant add-in was used for morphological analysis. Quantification of neurite numbers is done be averaging the number of neurites per cell in the observed area from all the samples collected. The software add-in can calculate the numbers automatically.

### Western Blotting

After electrical stimulation, cells were harvested with lysis buffer (containing NP40, Protease Inhibitor Cocktail, 1 mM PMSF and 0.5 mM β-glycerophosphate). Total protein concentrations were determined by BCA Assay (Thermo Fisher Scientific, NSW), and detected using a SpectraMax Plus384 absorbance microplate reader (Molecular Devices, Sunnyvale, CA). Samples were heat-treated in Laemmli buffer at 95 ^o^C, loaded to 8% SDS-PAGE gels for fractionation, and then transferred onto Immun-BlotTM PVDF membranes (Bio-Rad, Hercules, CA). The block consisted of 5% BSA in TBST. The membranes were then incubated with synaptophysin, PSD95 (Thermo Fisher Scientific, NSW; dilution factor 1:1000), and PSA-NCAM (Thermo Fisher Scientific, NSW; dilution factor 1:1000) antibodies in TBST containing 1% BSA overnight at 4 ^o^C. Secondary antibodies were anti-rabbit IgG conjugated with horseradish peroxidase (Santa Cruz Biotechnology, Santa Cruz, CA; dilution factor 1:5000). For visualization, ECL detection reagents were exposed on the GelDoc System (Bio-Rad, Hercules, CA). Images were analysed using ImageJ 1.46r software (NIH, USA).

### RT-qPCR

The RT-qPCR protocol was adopted from our previous work^5,22^. Briefly, total RNA was extracted using the PureLink RNA extraction kit (Thermo Fisher Scientific, NSW) according to the manufacturer’s protocol. First-strand cDNA was synthesized with the VILO cDNA synthesis kit (Thermo Fisher Scientific, NSW) with 20 μL reaction volume. qRT-PCR was carried out in triplicate using SYBR Select Master mix (Thermo Fisher Scientific, NSW) on a C1000 Touch Thermal Cycler coupled with CFX96 Real-Time System (Bio-Rad, Hercules, CA). The results were normalized to mouse GAPDH, and were expressed as fold different from controls. The primers for the target genes (Synaptophysin, PSD95, NCAM) as well as internal control (GAPDH) are detailed in Supplementary Material.

### Statistics

SPSS (version 15; Chicago, IL) was used for statistical analysis. One-way analysis of variance (ANOVA) or two-way ANOVAs with post-hoc Tukey’s tests were performed for multiple comparisons. Data were expressed as mean ± SEM, and *p* < 0.05 was considered statistically significant.

## Results

### Electrochemical properties of the PPy.DBSA coated electrodes

**Figure 3:**
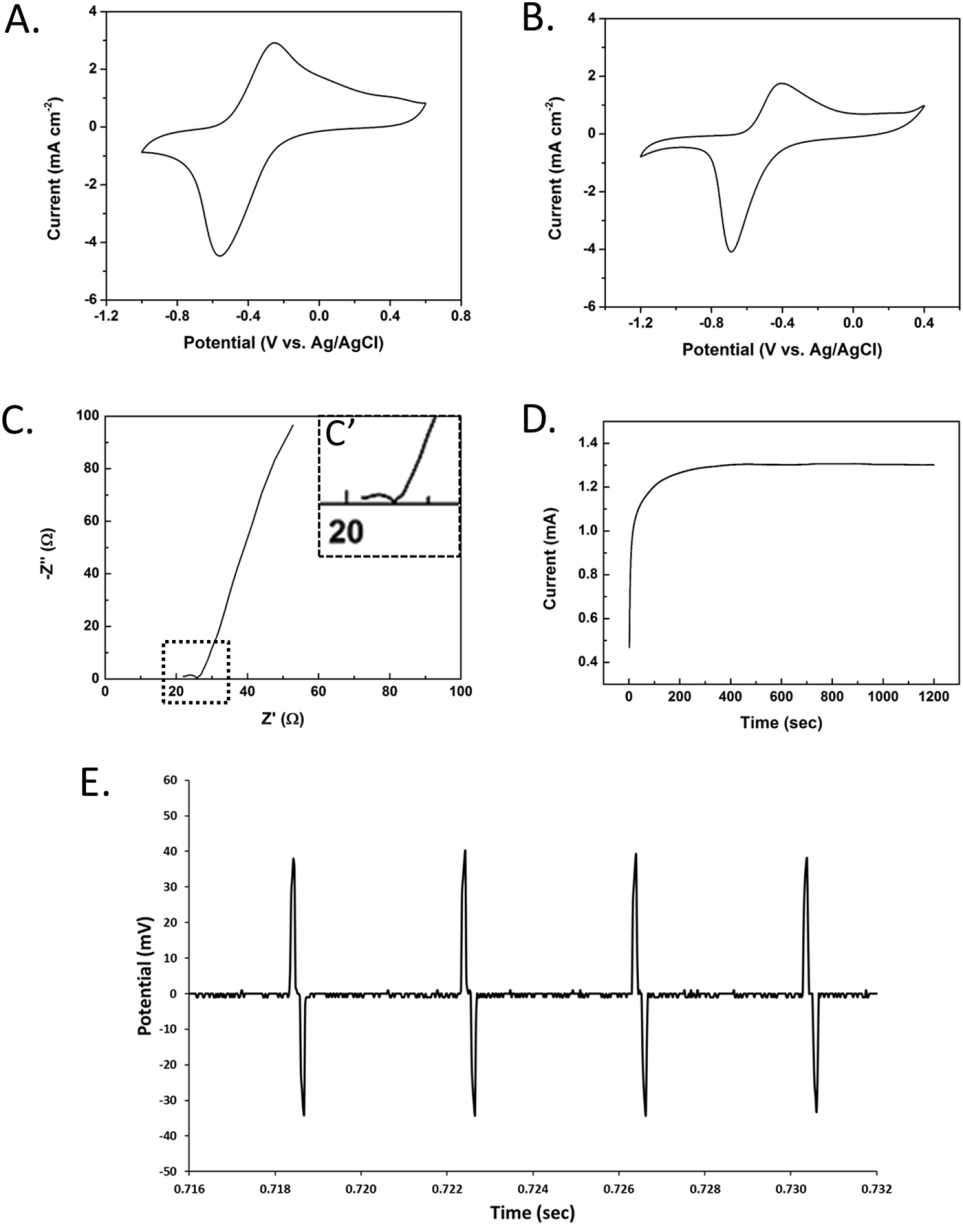
Electrochemistry properties of interdigitated 3D electrodes. (**A**) CV curves of PPy coated 3D electrode in PBS at 10 mV s^−1^ (vs. Ag/AgCl); (**B**) CV curves of PPy coated 3D electrode in collagen at 10 mV s^−1^ (vs. Ag/AgCl); (**C**) Nyquist plot of PPy coated 3D electrode in PBS (C’: insert demonstrates the compressed semicircle, indicating low charge transfer resistance, ~2.5Ω); (**D**) Chronoamperograms for PPy growth on 3D electrode; (**E**) Voltage waveform when biphasic current (1 mA cm^−2^) pulses were applied on PPy coated 3D electrodes in collagen (after 8 h stimulation). We use a symmetric configuration consists of two 3D electrodes as shown in Fig. 2A to finish the stimulation test. Following our previous publication (Xiao *et al*.^25^), a biphasic 250 Hz pulsed current was applied to the cell and the resulting voltage was recorded.

The 3D interdigitated electrodes (Fig. 1) were cleaned by sonication in acetone and deionized water. Polypyrrole (PPy) was deposited on the electrode using electropolymerization induced at a constant potential. Figure 3D shows a characteristic chronoamperogram obtained during PPy growth. The initial increase in current with time is indicative of polymer deposition with increasing surface area of the electrode developing with time.

Figure 3A,B shows the cyclic voltammograms obtained in PBS using the 3D coated electrodes with or without collagen. As expected, a redox couple appeared at around −0.6 V and −0.4 V, and this can be attributed to PPy oxidation and reduction^23^. Here the DBS ions are not directly involved in the redox process because the dopant anion is trapped inside the polymer matrix^24^, and so charge balance is achieved through ingress and egress of cations. The Nyquist plot obtained for these electrodes exhibited a compressed semicircle in the high frequency region and a line in the low frequency region (Fig. 3C), with low charge transfer resistance (~2.5 Ω, diameter of the semicircle).

Two interdigitated 3D electrodes were assembled into a device and filled with collagen (without living cells). The device was then subjected to a charge-balanced biphasic 250 Hz pulsed current stimulation^25^. Figure 3D shows a representative chronoamperograms for Ppy growth on the 3D electrode, while Fig. 3E shows a representative example of the resulting voltage waveform when biphasic current pulses were applied to PPy coated on 3D electrodes. The results obtained indicate that the collagen media is capable of providing sufficient ionic conductivity to allow efficient charge transfer during current pulsing.

### 3D electrical stimulation improved neurite growth in wild-type and NRG1-KO mice

We have previously reported that electrical stimulation using PPy.DBSA as an electrode conduit can improve neurite growth in 2D^10^. To confirm this effect in 3D, we used the same stimulation regime (1 ± 0.25 mA/cm^2^ stimulated over 8 h/day for 3 consecutive days) and culture conditions, but conducted the electrical stimulation on primary neurons encapsulated in 3D collagen gels, and stimulated with our 3D interdigitated electrodes.

**Figure 4:**
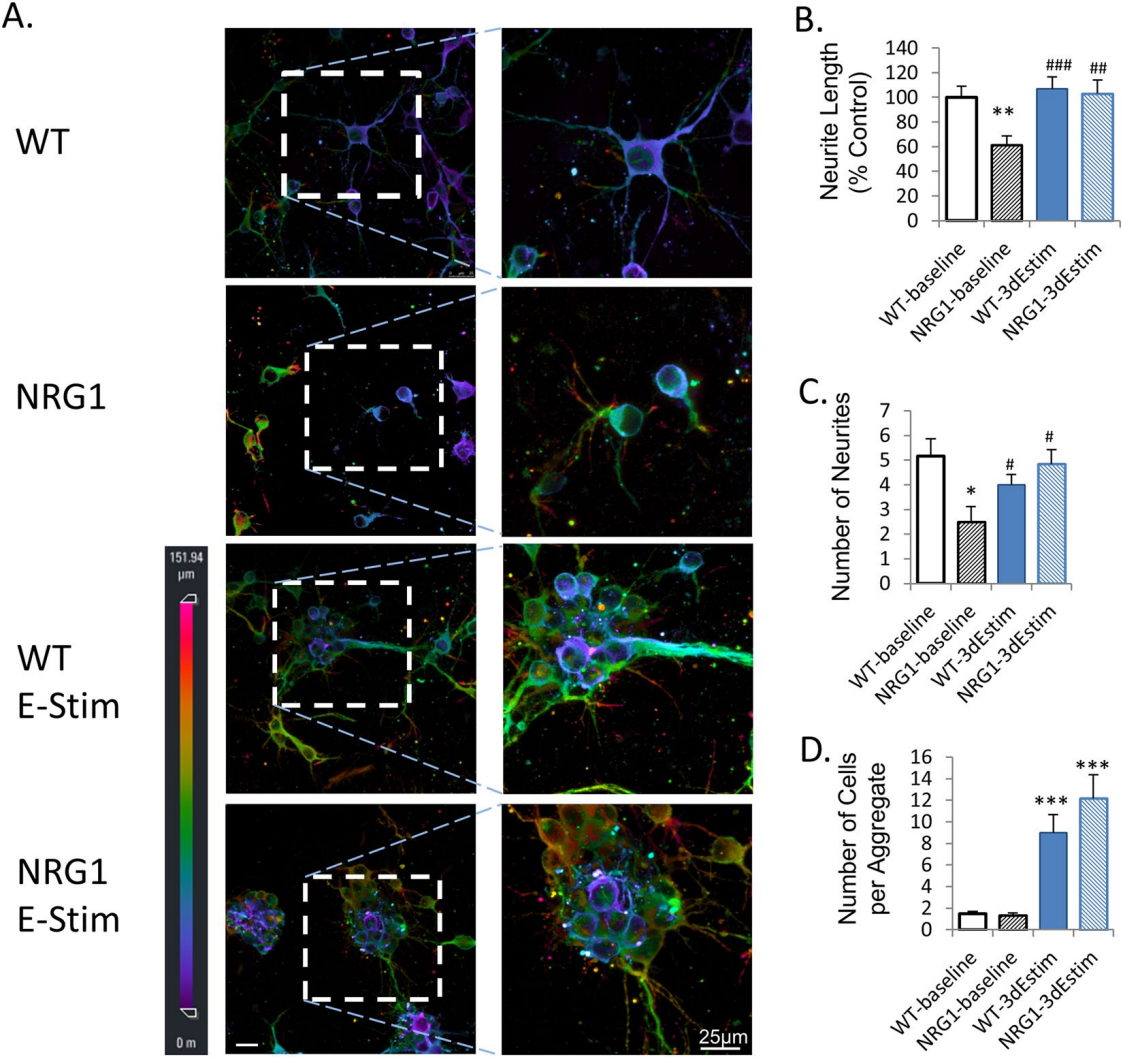
Effects of 3D electrical stimulation on cell morphorlogy of primary PFC neurons in collagen (detected by MAP2 immunofluorescence in 3D confocal imaging). Scale bar = 25 µm. *p < 0.05 vs wild-type, baseline; **p < 0.01 vs wild-type, baseline; ***p < 0.001 vs wild-type, baseline; ^#^*p* < 0.05 vs NRG1-KO, baseline; ^##^*p* < 0.01 vs NRG1-KO, baseline; ^###^*p* < 0.001 vs NRG1-KO, baseline. Error bars indicate SEM.

As shown in Fig. 4, both the neurite length and number of neurites was significantly reduced in NRG1-KO neurons compared to those from wildtype in a 3D culture (*P* < 0.01 and *P* < 0.05, respectively). As expected, these reductions were rescued by 3D electrical stimulation with PPy.DBSA (*P* < 0.01 and *P* < 0.05, respectively).

Interestingly, when electrical stimulation was induced in 3D structures, cell aggregates and neurite bundles were observed, which is significantly different from non-stimulated cells (Fig. 4A,D). These effects were not observed in previous studies using 2D cultures^10^.

### 3D electrical stimulation improved expression of markers for synaptogenesis

**Figure 5:**
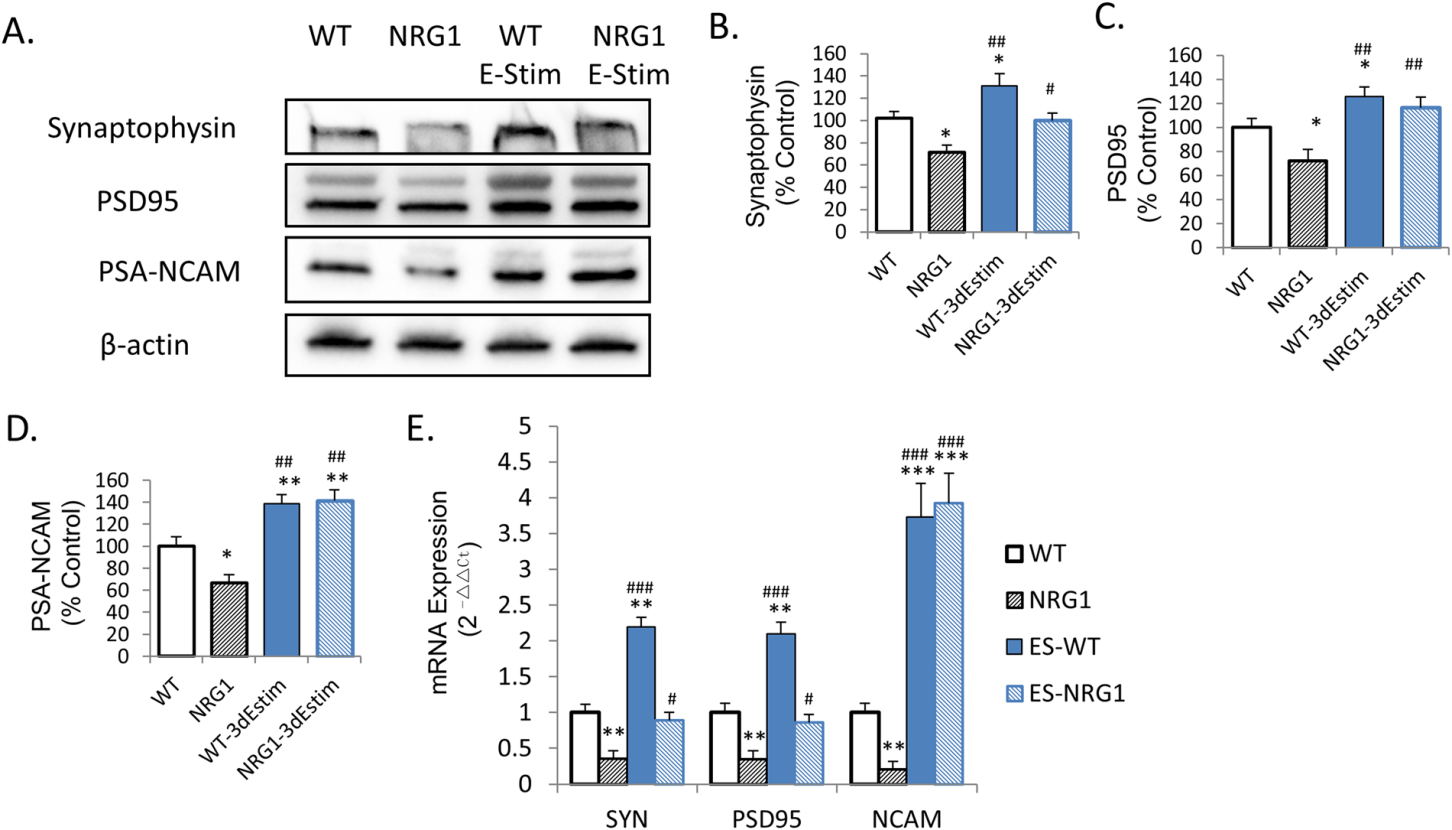
Effects of 3D electrical stimulation on protein and mRNA expression of Synaptophysin, PSD95 and PSA-NCAM. *p < 0.05 vs wild-type, baseline; **p < 0.01 vs wild-type, baseline; ***p < 0.001 vs wild-type, baseline; ^#^*p* < 0.05 vs NRG1-KO, baseline; ^##^*p* < 0.01 vs NRG1-KO, baseline; ^###^*p* < 0.001 vs NRG1-KO, baseline. Error bars indicate SEM.

Western blotting revealed that protein expression of the pre-synaptic marker synaptophysin and the post-synaptic marker PSD95 was decreased in 3D neuronal cultures of NRG1-KO mice, as compared to those from wildtype. Interestingly, 3D electrical stimulation with PPy.DBSA increased protein expression of these markers (Fig. 5), hence preventing the reduction of synaptogenesis induced by NRG1-KO.

Similarly, results from RT-qPCR suggested that mRNA levels of synaptophysin and PSD95 was decreased in NRG1-KO 3D neuronal cultures, as compared to their wildtype counterparts. These reductions were rescued by 3D electrical stimulation (Fig. 5).

### 3D electrical stimulation improved neural cell aggregation

Immunofluorescence of MAP2 antibody revealed that 3D electrical stimulation can lead to cell aggregation, demonstrated with the increased number of cells per aggregate (Fig. 4). There was no significant difference between the different genotypes in cell aggregation. The effect of 3D electrical stimulation on cell aggregation was confirmed by western blotting of a cell aggregation marker (NCAM), which revealed that although PSA-NCAM was reduced by NRG1-KO, 3D stimulation had increased the protein expression of this marker to a level even higher than the wild-type control (Fig. 5). Similar effects were also observed in mRNA levels of NCAM illustrated by RT-qPCR (Fig. 5).

### Comparison between 2D and 3D culture

**Figure 6:**
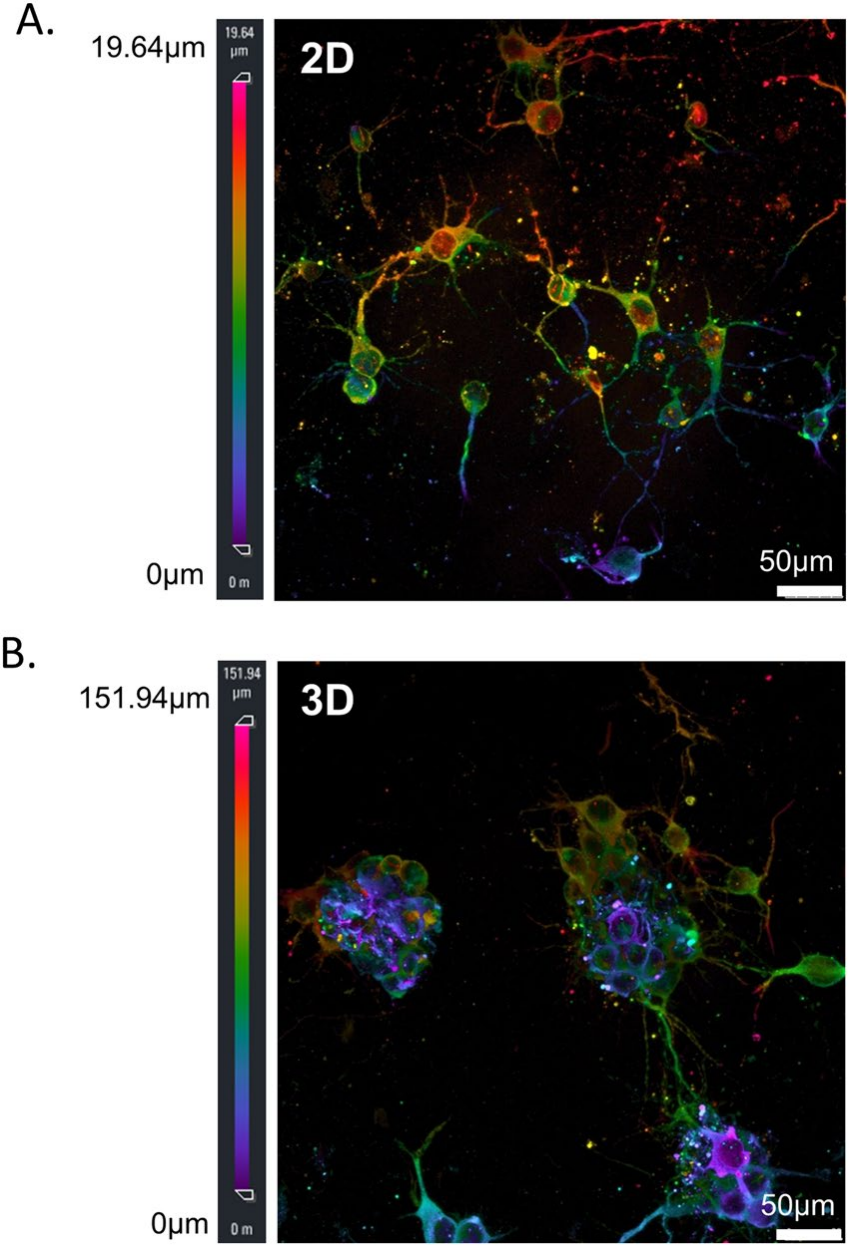
2D culture vs. 3D culture after electrical stimulation. (**A**) In 2D culture, primary PFC cells were randomly located as individual cells, even after electrical stimulation. These neurons communicate with each other with individual neurites. (Z stack depth = 19.64 μm; scale bar = 50 μm). (**B**) In 3D culture, electrical stimulation triggered cell aggregation. PFC neuronal cells tend to stick together forming aggregates, communicating with nearby aggregates by bundles of neurites. (Z stack depth = 151.94 μm; scale bar = 50 μm).

In order to provide a concise comparison between 2D and 3D culture under electrical stimulation, we used immunofluorescence of MAP2 to reveal the morphological differences between the two systems. As shown in Fig. 6A, electrical stimulation on 2D cell culture did not promote cell aggregation, as we have previously shown in our past publication^10^; while electrical stimulation on 3D cell culture promote cell aggregation, and the neurites from the same cell aggregate tended to form bundles before projecting to a neighbouring aggregate (Fig. 6B).

## Discussion

To the best of the authors’ knowledge, this is the first report demonstrating the effect of 3D electrical stimulation on neurite outgrowth in primary neural cultures from both normal and disease models of schizophrenia. The morphological data from immunofluorescence of MAP2, as well as the protein and mRNA expression data suggest that the 3D primary neuronal cultures established in the present study represent the disease phenotype of schizophrenia as we observed previously in 2D^2,10^, while providing extra biological insights in terms of cell aggregation and 3D neurite outgrowth (Figs 4A,D and 5).

Additive fabrication, commonly known as 3D printing, is a layer by layer production method that is enabling advances in both research and industry. Additive fabrication allows for design flexibility that cannot be readily achieved through conventional manufacturing processes. Objet Polyjet technology (Stratasys, USA) enables the production of components from UV curable polymers with layer resolution of 16 µm. Selective Laser Melting (SLM) is a metal-based additive technique with layer resolution of 25 µm. This process controls the application of a focussed high energy laser to a levelled bed of metal powder, elevating the temperature of the powder beyond its melting point at discrete locations to form dense welds, and is ideally suited to the production of complex electrode geometries.

The interdigitated 3D electrode allowed for electrical stimulation to be conducted through the 3D collagen cell culture. The electrochemistry characterization tests (Fig. 3) revealed that the electrochemistry properties of the interdigitated 3D electrodes were as expected. This is critical for the evaluation of biological effects of stimulation via these electrodes. Moreover, the technique of 3D electrical stimulation in collagen-encapsulated cell culture enables the evaluation of electrical stimulation in an environment more closely aligned with the *in vivo* environment, allowing for free movement of cells and neurite growth within the 3D hydrogel without the restriction of 2D substrates^26,27^. Without the restriction of movement, the cells in 3D developed aggregates and neurite bundles, indicating enhanced movement of cells and improved communication. The main advantage of this system is that it is a REDOT driven stimulation system by putting the electrodes into real contact with the cells in 3D (not merely an electric field effect, as in the 2D electrode systems used in our and others’ previous studies^6,8,10^). With minimum optimization, this system could be translated into clinical settings as a possible treatment option for patients with developmental mental illness (such as schizophrenia).

The results of neurite outgrowth in the present study suggest that this 3D primary neuronal culture is a reliable *in vitro* model of the disease model of neurite outgrowth deficit derived from the primary cultures of the NRG1-KO mice. This was evaluated by the morphological study, as well as the protein and mRNA expressions of the pre-synaptic and post-synaptic markers synaptophysin and PSD95, respectively.

NRG1-KO is regarded as one of the key genetic susceptibility factors for neurodevelopmental deficits in schizophrenia and related disorders^28^. In the present study, both neurite length and number of neurites are reduced by NRG1-KO compared to the wild-type, which is consistent with our previous findings in 2D cell culture of these neuronal cells^2,10^. Similarly, the protein and mRNA expressions of synaptophysin and PSD95 were also reduced by NRG1-KO in 3D cell culture as compared to wild type. These deficits were rescued by 3D electrical stimulation with the biocompatible conducting polymer PPy.DBSA, indicating that this 3D primary cell culture platform can not only be used as a platform to mimic the biological functions as well as morphological changes in NRG1-KO models of deficits in neurite outgrowth and synaptogenesis, but it can also be used as a testing system for the effect of 3D electrical stimulation on this disease model.

The expression of NCAM in the brain is important in the regulation of many neurodevelopmental processes, including cell-cell adhesion, neurite outgrowth, and synaptic plasticity^29^. PSA-NCAM expression was reduced in layers IV and V of dorsolateral prefrontal cortex in schizophrenia patients compared to the control^30^. Moreover, NCAM-null mice have defected mossy fibre laminations^31^. It has been suggested that decreased PSA-NCAM may be indicative of a diminished capacity for synaptic plasticity and remodelling in schizophrenia^32^. In the present study, PSA-NCAM protein and mRNA expression was found to be decreased in primary PFC neuronal cultures of NRG1-KO mice compared to wildtype, supporting the aforementioned hypothesis. In addition, 3D electrical stimulation increased the expression of PSA-NCAM to a level that was significantly higher than the non-stimulated wildtype neurons. Since biosynthesis of PSA-NCAM is regulated by cell activation such as electrical activity in axons^33^, the increased expression of PSA-NCAM under 3D electrical stimulation observed in the present study may be due to the increased electrical activity of axons of these neurons triggered by electrical stimulation. This needs to be further investigated in future studies.

NCAM and PSA-NCAM have also been suggested to interact with a number of molecules and signalling pathways including BDNF-TrkB signalling. Decreased BDNF signalling mediates PSA-dependent defects in long-term potentiation^34^. We have previously reported that BDNF signalling was downregulated in primary neuronal cultures of NRG1-KO mice, but rescued by electrical stimulation. Therefore, the interaction between PSA-NCAM and BDNF signalling may play an important role in mediating the effect of electrical stimulation in promoting neurite outgrowth and synaptic plasticity as observed in the present study.

Finally, the increase in expression of pre- and post-synaptic markers can merely be a result of enhanced cells interactions, which results from their aggregation, although we have observed similar effects in our 2D experiments without the presence of cell aggregation. Further study is required to isolate the effect of aggregation and direct effect of electrical stimulation. For example, a cell aggregation inhibitor might be added to the culture media to halt the aggregation during electrical stimulation. The delivery of electrical stimulation by the 3D interdigitated electrode system in 3D cell culture provides a synergistic effect of 3D cell culture and 3D electrical stimulation. Future studies are warranted to investigate the unique attributes of the two. Due to limited tissue availability, this project only provides a proof of concept for the 3D electrical stimulation effects on neurite growth based on our previously tested electrical stimulation parameter. Further studies to test the various effects of electrical stimulation from this device based on various stimulation parameters are also warranted.

In conclusion, the interdigitated 3D electrodes enable the delivery of electrical stimulation though the 3D primary cell culture encapsulated in the collagen gels, which represented the morphological and molecular biological characteristics of the neurite deficits and synaptic deficits in the NRG1-KO model *in vitro*. This 3D electrical stimulation system provides a platform for rescuing the neurite outgrowth deficits in a 3D culturing environment, one similar to the *in vivo* biological system. The observation of cell aggregates as well as the upregulated PSA-NCAM protein and transcript expression suggest a possible pathway which may play an important role in the effect of 3D electrical stimulation in prompting neurite outgrowth and synaptic plasticity. Although further studies confirming the exact mechanism of 3D electrical stimulation on these effects are required, this study provides a new concept for not only a possible diagnostic platform for neurite deficits in neurodevelopmental diseases such as schizophrenia, but also a viable platform to test various treatment options (such as drug delivery through the gel or on the surface of the 3D electrodes) in combination with electrical stimulation or on its own. It also challenges the validity of 2D studies in this area.
